# The Tragedy and Comedy of the Act

**Published:** 1914-02-21

**Authors:** 


### THE PROFESSION OF MEDICINE.

### THE TRAGEDY AND COMEDY OF THE ACT.

It is probable that no Act of Parliament has ever been so fruitful of deep tragedy on the one hand or so ripe for ridicule on the other. Tragedies there are every day in plenty. A panel system which, apart from inherent evils of cheap contract service, lock-up surgeries, lay control (often uneducated at that), and a limited drug fund, repels half the medical profession, while it opens the door to unqualified practitioners, bone-setters, "cancer curers," and the like, has familiarised the public not only with the "panel doctor," but with the "panel inquest" as well.

Then there is the tragedy of the sanatorium benefits, inadequate or at a standstill in many of our large towns; hopeless of attainment in others. Patients fully insured are returned to die at home, or, worse still, in Poor-Law infirmaries, where, as State-manufactured paupers, they have become a further burden to over-taxed ratepayers.

There is tragedy, too, in the knowledge that while the Insurance Act has brought about a decrease in the charitable contributions to hospitals, there has been no perceptible diminution in the demands made upon them as regards admissions of insured persons; and this is especially significant in the case of Poor-Law infirmaries. The Insurance Act, indeed, has been described, not inaptly, as the "tombstone of philanthropy"; but it might equally well be termed the "whetstone of wit," for just as it has enlarged the national vocabulary—not always, it is true, in a desirable direction—so also it has sharpened the national sense of humour, and provided the stage comedian and the raconteur of the smoking-room with many a "panel" yarn or smart Insurance epigram.

One of these was rescued from oblivion and recorded by the *Daily Chronicle* in its account of the complimentary dinner given to Dr. Addison by the panel doctors and is worth reproduction. A speaker, in supporting the toast of the guest of the evening, is reported to have said that "a great many of those who did not exactly bless it when it started, remained to pray; and, as Mr. Lloyd George had told them, to take part in the collection." The phrase precisely defined the two principal motives which it is to be feared had impelled a very considerable number of the audience to turn their backs on their pledges and their profession. As far as the incident went, it was proved that even in this artificial age of political philanthropy, joy-days, and Insurance feasts, truth is on occasion to be sometimes found at the bottom of a champagne glass.

